# Aggressive natural killer-cell leukemia with jaundice and spontaneous splenic rupture: a case report and review of the literature

**DOI:** 10.1186/1746-1596-8-43

**Published:** 2013-03-11

**Authors:** Li-min Gao, Wei-ping Liu, Qun-pei Yang, Hui-fang Li, Jun-jie Chen, Yuan Tang, Yan Zou, Dian-Ying Liao, Yan-mei Liu, Sha Zhao

**Affiliations:** 1 Department of Pathology, West China Hospital of Sichuan University, Chengdu 610041, China; 2 Laboratory of Stem Cell Biology, State Key Laboratory of Biotherapy, West China Hospital of Sichuan University, Chengdu 610041, China; 3 Department of Burns and Plastic Surgery, West China Hospital of Sichuan University, Chengdu 610041, China

**Keywords:** Aggressive natural killer-cell leukemia, Jaundice, Spontaneous splenic rupture

## Abstract

Aggressive natural killer cell leukemia/lymphoma (ANKL) is a rare aggressive form of NK-cell neoplasm. We report an uncommon case of 36-year-old male who showed jaundice and spontaneous splenic rupture. The diagnosis was established by the biopsy of liver and spleen. The monomorphous medium-size neoplastic cells infiltrated into portal areas and sinus of liver as well as the cords and sinus of the spleen. Necrosis, mitotic figures and significant apoptosis could be seen easily. These neoplastic cells demonstrated a typical immunophenotype of CD3ε+, CD56+, CD16+, Granzyme B+, TIA-1+. T-cell receptor γ (TCR-γ) gene rearrangement analysis showed germline configuration and the result of in situ hybridization for Epstein-Barr virus-encoded RNA (EBER-ISH) was positive. The patient has undergone an aggressive clinical course and died of multi-organ function failure 14 days later after admission. To the best of our knowledge, this is the first case of ANKL with spontaneous splenic rupture, and we should pay more attention to recognize it.

**Virtual Slides:**

The virtual slide(s) for this article can be found here: http://www.diagnosticpathology.diagnomx.eu/vs/2048154883890867

## Background

Aggressive natural killer cell leukemia/lymphoma (ANKL) is a rare neoplasm which comprises less than 0.1% of all lymphoid neoplasms [1]. Different from the usual leukemia, the neoplastic cells in ANKL can be sparse in peripheral blood and bone marrow [2]. From our review of relevant literature, the patient we reported is the first case of ANKL with spontaneous splenic rupture as the initial symptom.

## Case presentation

### Case report

**Figure 1:**
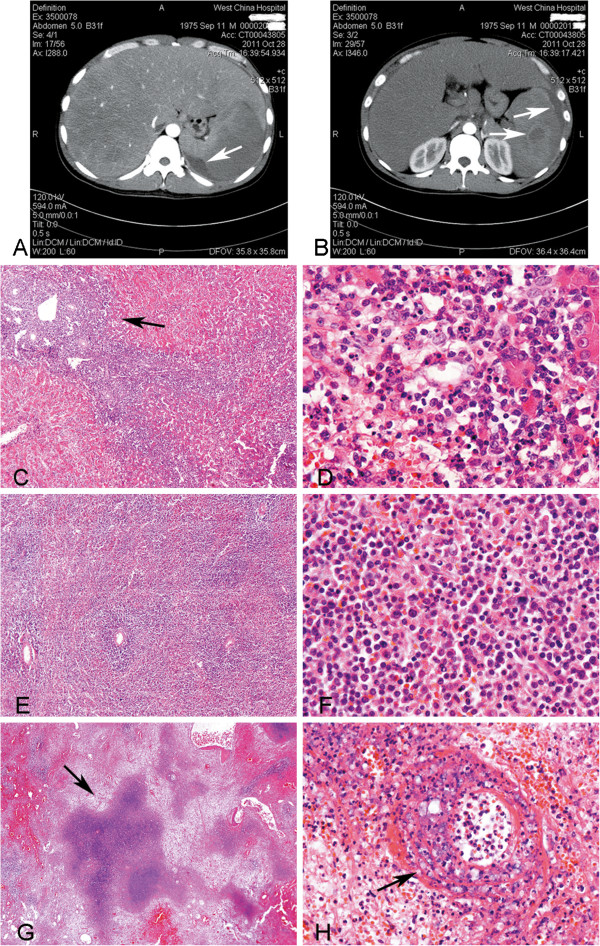
**Hepatosplenic CT images and histopathology.** (**A**, **B**) CT scan demonstrated hepatosplenomegaly; hemorrhage was observed in splenic parenchymal and perisplenic (arrow), the largest hematoma was located under the spleen and the maximum cross-sectional area of which was 9.5 cm × 4.3 cm; ascites and enlarged lymph nodes of peritoneal cavity were also noted. (**C**) Histologic examination of liver showed the portal areas and sinusoidal infiltration (arrow) (H&E, original magnification × 40). (**D**) Neoplastic cells in the liver were monomorphic and medium size with irregular nuclei. Mitotic figures and apoptosis can be easily seen (H&E, original magnification × 400). (**E**) Neoplastic cells in the spleen were observed in cords and sinuses of red pulp, as well as around arteriolar sheath (H&E, original magnification × 40). (**F**) Neoplastic cells in the spleen were monomorphic and medium sized (**H** &**E**, original magnification × 400). (**G**) Necrotic areas were seen in the spleen (arrow) (**H** &**E**, original magnification × 40). (**H**) The blood vessel infiltration phenomenon was observed in the spleen (arrow) (**H** &**E**, original magnification × 264).

A 36-year-old man had presented with jaundice and pain of abdomen for 5 days. The patient seemed to get a cold 5 days ago before he was sent to hospital, then a serious jaundice of general skin appeared, accompanying with a bursting pain on the middle abdomen, which was persistent without reflection. There were some other symptoms such as nausea, chest tightness, muscle weakness and anorexia. The second day after admission, the patient had a significant pain on the upper abdomen, with rebound tenderness. Physical examination showed no palpable superficial lymph nodes. His abdomen was soft, while light tenderness was presented in the upper abdomen without rebound tenderness. The liver was palpable below the right costal margin, but the spleen was impalpable. Laboratory tests demonstrated (1) White blood cells 4.60 × 10^9^/L (neutrophil 84.7%, lymphocyte 10.3%, no abnormal cells had been found), red blood cells 3.87 × 10^12^ L, platelets 64 × 10^9^/L. hemoglobin 140 g/L (2) Total bilirubin 340.5 umol/L, direct bilirubin 281.0 umol/L, aspartate aminotransferase 1163 U/L, glutamic-oxal(o) acetic transaminase 1765 U/L, lactate dehydrogenase 1253 IU/L, total bile acid 109.4 umol/L.(3) Prothrombin time 27.2 s, activated partial thromboplastin time 43.8 s, fibrinogen 1.36 g/L, thrombin time 26.4 s. Computer tomography scans revealed hepatosplenomegaly; hemorrhage was observed in splenic parenchymal and perisplenic, the largest hematoma was located under the spleen and the maximum cross-sectional area of which was 9.5 cm × 4.3 cm; ascites and enlarged lymph nodes of peritoneal cavity were also noted. [Figure 1(A, B)] Splenic rupture was diagnosed by CT scans. Splenectomy was performed immediately and the biopsy of liver was implemented simultaneously.

### Pathologic findings

**Figure 2:**
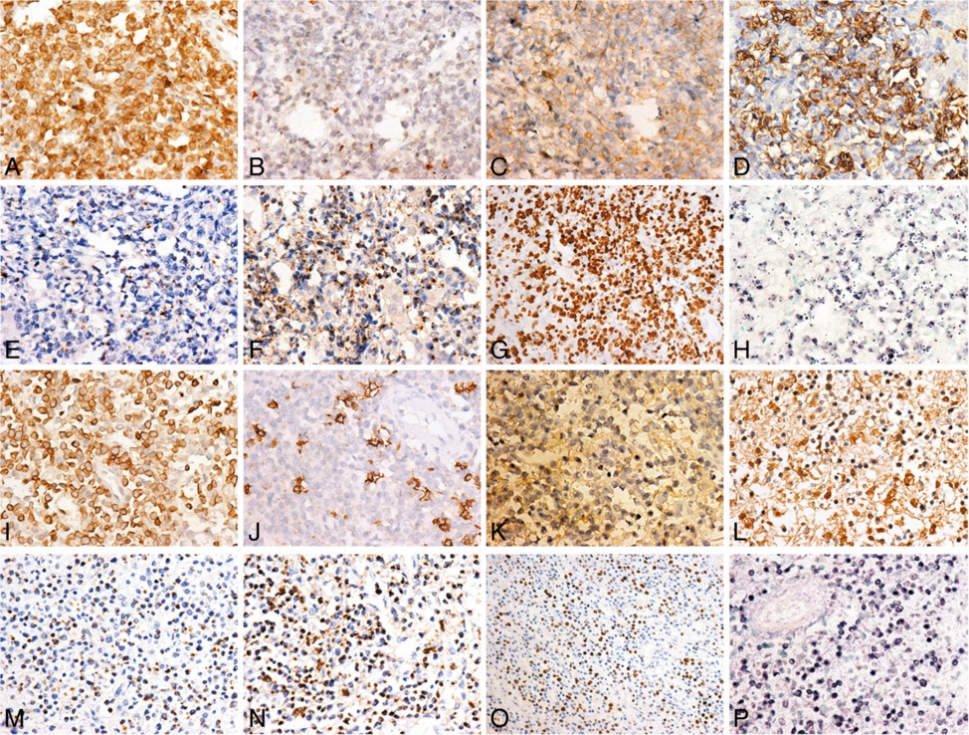
**Immunohistochemistry staining of the hepatic tissue and splenic tissue.** The tumor cells were positive for CD3ε (**A**, liver; I, spleen. original magnification × 400), negative for CD20 (**B**, liver; **J**, spleen. original magnification × 400), and they were positive for CD56 (**C**, liver; **K**, spleen. original magnification × 400), CD16 (**D**, liver; **L**, spleen. original magnification × 400) Granzyme B (**E**, liver; M, spleen. original magnification × 400), TIA-1 (**F**, liver; **N**, spleen. original magnification × 400)., the Ki-67 index was about 90% **G**, liver; **O**, spleen. original magnification × 200). The result of EBER-ISH was positive (**H**, liver; **P**, spleen. original magnification × 400).

**Figure 3:**
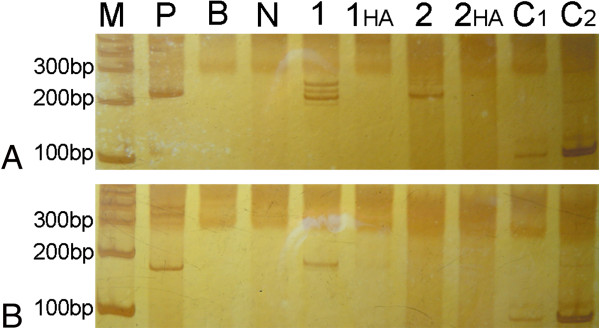
**Polymerase Chain Reaction heteroduplex analysis.** No obvious band of cloned TCR-γ gene rearrangement was detected PCR-HA (**A**, **B**),(M, maker; p, positive control; B, blank; N, negative control; 1, liver; 2, spleen; HA, heteroduplex analysis; C1, internal control of liver; C2, internal control of spleen).

Macroscopically, a small piece of tissue was taken from the liver for biopsy and the volume was 1.5 cm × 0.8 cm × 0.6 cm. It was grey-brown in color with smooth capsule. Spleen was dissected completely with 19.0 cm × 12.5 cm × 7.0 cm in volume and 870.0 gram in weight. The capsule of splenic hilum was absent and lots of blood clots were seen in this area. A tremendous subcapsular hematoma was observed after the spleen was cut in slices. Microscopically, the normal structure of the liver was partly damaged, and many monomorphic medium-sized cells infiltrated into portal areas and sinusoids, with thin to moderate rim of pale or amphophilic cytoplasm, irregular nuclei, slightly condensed chromatin and inconspicuous nucleoli. Mitotic figures and apoptosis were obvious in these areas [Figure 1(C, D)]. The sections from the spleen showed expansion of the red pulp with relative depletion of the white pulp. Neoplastic cells were found in the cords and sinuses of red pulp, as well as around splenic arteriolar sheath. The morphology and size of tumor cells were similar to which infiltrated into the liver [Figure 1(E, F)]. Necrosis and the blood vessel infiltration phenomenon could be observed in the spleen [Figure 1(G, H)]. Immunohistochemically, the neoplastic cells were CD3ε+, CD56+, CD16+, CD43+, Granzyme B+, TIA-1+, CD20-, CD2-, CD5-, CD7-, CD4-, CD8- and CD123-. The proliferation index was approximately 90%, assessed by Ki-67 staining and the result of EBER-ISH showed positive for most of the abnormal cells (Figure 2). No obvious band of cloned TCR-γ gene rearrangement was detected by the Polymerase Chain Reaction heteroduplex analysis (PCR-HA) and polyacrylamide gel electrophoresis (PAGE) (Figure 3). Combined with morphology and the result of immunohistochemistry, TCR-γ gene rearrangement analysis and in situ hybridization, the final diagnosis was made as aggressive natural killer cell leukemia/lymphoma (ANKL).

### Follow up

The patient has undergone an aggressive clinical course and developed into multiple organ dysfunction syndromes (MODS) in a short time. The patient died of multi-organ function failure 14 days later after hospitalization. Without the agreement of his family, the autopsy was not performed.

## Discussion

ANKL is a rare form of neoplasm characterized by a systemic proliferation of NK cells with an aggressive clinical course. It is originally recognized in the mid-1980s as a non-T-cell type of aggressive large granular lymphocyte (LGL) leukemia [3]. This disease has a higher incidence in Asians compared with other ethnic populations. Most of the patients are young people, with a median age of 42 years old, men and women are equally affected or men have a slight predominance [4]. NK neoplastic cells are almost invariably infected with Epstein-Barr virus (EBV), and it is suggesting that the virus may be of pathogenetic significance [5]. ANKL mainly involved in bone marrow and peripheral blood, as well as liver and/or spleen. Skin involvement is rare. Patients are typically very ill with fever, anemia, thrombocytopenia, disseminated intravascular coagulation (DIC) and liver function disturbances [5,6]. High levels of serum lactate dehydrogenase can also be found in the serum. Liver function derangement, disseminated intravascular coagulopathy and multiorgan failure appear progressively and most patients die within days to weeks after presentation, with a median survival of 2 months [1].

About 150 cases have been reported in the literature and the largest series is 22 cases analyzed by R Suzuki, et al. [3]. Among those cases, 107 (71%) with spleen involvement and 109 (72%) with liver involvement. Major clinical manifestations included hepatosplenomegaly, jaundice and liver function disturbances, while there is no report of splenic rupture. So our case is the first report of ANKL complicating with spontaneous splenic rupture. The patient we report is 36 years old and the main symptoms are sudden jaundice and hepatosplenomegaly. The unique feature in this case is its presentation as spontaneous (pathologic) splenic rupture. The mainly possible causes of spontaneous splenic rupture in this kind of leukemia are suspected as follows: 1. Most commonly, malignant cells infiltrate into the spleen directly and their sheer volume exceeds the capacity of the relatively nondistensible splenic capsule, causing capsular rupture, and splenic hemorrhage. 2. Splenic infarction with vascular invasion, consequent subcapsular hemorrhage and subsequent rupture of splenic capsule. 3. Coagulation disturbance related to the disease [7]. This reported case highlights the importance of considering diagnosis of ANKL in patients presenting with jaundice, massive hepatosplenomegaly and the possibility of splenic rupture.

As reported, different from the usual leukemia, the leukemia cells of ANKL constitute less than 5% to more than 80% of lymphocytes in the peripheral blood, and account for 6% to 92% of nucleated cells in the bone marrow [2], so it is appropriate to term this neoplasm as “leukemia/lymphoma” [2]. In most reported cases in the literature, the diagnosis of ANKL is based on the result of bone marrow aspiration and/or the peripheral blood smear, and it is seldom mentioned on the biopsies on the liver and spleen [8]. However, as we all known the characteristic of this leukemia is that the neoplastic cells in ANKL can be sparse in peripheral blood and bone marrow, so the ANKL can be diagnosed by neoplastic cells existing in spleen and liver without the evidence in the peripheral blood and/or bone marrow. Our reported case has some features as following: First, the patient has showed a highly aggressive clinical course with massive hepatosplenomegaly, splenic rupture, coagulation disorders and short survival time, no history of other disease. Then a diffuse and destructive infiltration of monomorphous neoplastic cells was observed in histologic sections of liver and spleen, which are medium in size, with few to moderate cytoplasm and irregular nuclei. Nuclei showed slightly condensed chromatin pattern and inconspicuous or distinct nucleoli. Necrosis, mitotic figures and significant apoptosis could be seen easily. Additionally, these neoplastic cells demonstrated a typical immunophenotype of CD3ε+, CD56+, CD16+, Granzyme B+, TIA-1+, CD43+. T-cell receptor γ (TCR-γ) gene rearrangement analysis showed germline configuration and the result of EBER1/2-ISH was positive. From above, this case was coincided with the pathologic diagnostic criteria for ANKL compared with previously reported cases [4,9,10]. Unfortunately, bone marrow biopsy and marrow smear of this patient had not been performed but the diagnosis of ANKL was still established according to the evidence of the biopsy of liver and spleen, clinical features and laboratory findings.

Extranodal NK/T-cell lymphoma, nasal type (ENKTCL-N) with advanced stage is also a highly aggressive neoplasm with a dismal clinical outcome, yet the relationship and boundary between ENKTCL-N with advanced stage and ANKL remains unclear [11]. There are many similarities between ENKTCL-N with advanced stage and ANKL, such as the morphology, immunophenotype, germline configuration of TCR gene and EBV association. However, Kwong, Y. L., et al. has pointed out ANKL can be different from ENKTCL-N by the absence of a previous history, a shorter illness, a younger age of presentation and an extremely aggressive course [5]. A recent array-based comparative genomic hybridization study indicates that loss of 7p and 17p and gain of 1q are frequent in ANKL which are different from ENKTCL-N [11], but the genetic changes still need to be proven by large amount case–control study.

ANKL Involving the liver and spleen can mimic the hepatosplenic T cell lymphoma which also presents jaundice and massive hepatosplenomegaly, but the neoplatic cells in hepatosplenic T cell lymphomas are commomly observed in sinus, and express T-cell makers, have rearranged TCR gene, and have no relatetion with EBV infection.

Systemic Epestein-Barr virus positive T-cell lymphoproliferative disease and ANKL also have some similarities: the fulminant clinical manifestations, presence of EBV in proliferating cells, and systemic hemophagocytosis. However, systemic EBV^+^ T-cell LPD is more common in children and monoclonal for T-cell receptor gene rearangement [2].

Plasmacytoid dendritic cell leukemia (pDCL) is a rare leukemia and needs to be identified with ANKL. Clinically, pDCL usually present an isolated cutaneous lesion at the time of diagnosis and rapidly evolves to multiple sites, proliferates into the blood, bone marrow, lymph nodes and other areas such as the spleen, liver, central nervous system (CNS),. Although expression of CD56, CD2, CD7 or granzyme B can be common between pDCL and ANKL, However, pDCL can be identified by the following phenotype: CD4±, high expression of CD123 (IL-3α receptor), BDCA-2 ± (blood dendritic cell antigen-2 or CD303±), BDCA-4 ± (CD304±), and it is not associated with EBV infection [12,13].

For other differential diagnosis, T cell lymphomas, such as peripheral T-cell lymphoma, not otherwise specified (PTCL-NOS) and anaplastic large cell lymphoma (ALCL) are also need to be considered. When tumor infiltrate into liver and spleen, clinical symptoms are similar with ANKL. Jaundice and massive hepatosplenomegaly are also can be seen. Meanwhile, morphologically, middle to large-sized neoplastic cells with atypia and mitosis can be detected in T cell lymphoma and ANKL. However, as previously mentioned, the characteristic T-cell makers, rearranged TCR gene, and have no relation to EBV infection are distinguishing features [14,15].

## Conclusion

This is the first case of ANKL with jaundice and spontaneous splenic rupture, which is hard to be diagnosed and easily confused with some other diseases. We should accumulate much experience to recognize it and treat it with effective methods for its fulminant clinical course.

## Consent

Written informed consent was obtained from the kin of the patient for publication of this case report and accompanying images. A copy of the written consent is available for review by the Editor-in-Chief of this journal.

## Abbreviations

ANKL: Aggressive natural killer cell leukemia/lymphoma; TCR-γ: T-cell receptor γ; EBER-ISH: In situ hybridization for Epstein-barr virus-encoded RNA; PCR-HA: Polymerase chain reaction heteroduplex analysis; PAGE: Polyacrylamide gel electrophoresis; MODS: Multiple organ dysfunction syndromes; LGL: Large granular lymphocyte; EBV: Epstein-barr virus; DIC: Disseminated intravascular coagulation; ENKTCL-N: Extranodal NK/T-cell lymphoma, nasal type; EBV+ T-cell LPD: Epestein-barr virus positive T-cell lymphoproliferative disease; pDCL: Plasmacytoid dendritic cell leukemia; BDCA: Blood dendritic cell antigen; PTCL-NOS: Peripheral T-cell lymphoma, not otherwise specified; ALCL: Anaplastic large cell lymphoma.

## Competing interests

The authors declare that they have no competing interests.

## Authors’ contributions

LG made contributions to acquisition of clinical data, and manuscript writing. SZ participated in its design and coordination and helped to draft and edit the manuscript. WL participated in design of the study and helped to confirm the diagnosis. QY, HL, JC and YL helped to manuscript writing, YT and YZ participated in molecular genetic studies, DL participated in immunoassays. All authors read and approved the final manuscript.
